# *Muscles*: An Overview of 2023 and Future Perspective

**DOI:** 10.3390/muscles3010001

**Published:** 2024-01-02

**Authors:** Corrado Angelini

**Affiliations:** Editor-in-Chief, MDPI AG, St. Alban-Anlage 66, 4052 Basel, Switzerland; corrado.angelini@unipd.it

Ending the year is an opportunity to reflect on the past twelve months. Without any doubt, we can say that the journal *Muscles* is growing wonderfully and successfully. Since 2022, the number of papers published has progressed: 17 publications in 2022 and 30 in 2023. A sufficient number of published articles was achieved in the three Special Issues of this past year, and others are on the way in the future.

Scientific journals form an essential part of academic and professional life, providing valuable insights into a wide range of subjects. In this Editorial, I will discuss the progress made in the field by the journal *Muscles*, exploring its growth, impact, and the significance of the increased number of publications within the discipline. By delving into this topic, we can gain a deeper understanding of the evolving nature of this journal and its relevance in shaping knowledge.

## 1. Evolution and Progress of *Muscles*

The journal *Muscles* has witnessed significant growth and evolution over the year 2023. In the past, traditional print journals provided a platform for disseminating knowledge. *Muscles* articles are mostly edited online by choice. The journal’s history tells that after the 2022 inaugural issue was released, *Muscles* transformed into a quarterly journal with technological advancements since online platforms emerged as the new medium for publishing and accessing scientific information.

This shift facilitates broader reach, increased reader engagement, and faster dissemination of information. Furthermore, incorporating multimedia elements, such as figures and interactive graphics, has revolutionized the way content is presented in our journal, enhancing the overall user experience.

## 2. Impact and Significance

*Muscles*, as a new journal, plays a crucial role in the academic and research community regarding neuromuscular disorders and muscle pathophysiology. The journal acts as a primary source of information, aiding scholars, researchers, and practitioners in staying updated with the latest advancements in these intriguing fields. By providing a platform for scholarly debates and critical analysis, this journal catalyzes intellectual growth and advancement. The journal fosters discussion, facilitates cross-disciplinary collaboration, and contributes to the overall progression of myology knowledge. The most successful and cited papers of last year were on using astaxanthin for muscle pain biomarkers after eccentric exercise [1], clinical gamma sarcoglycan’s socioeconomic impact [2], the role of mitochondria in muscle repair and longevity [3], and the use of muscle MRI in the diagnosis of limb-girdle syndromes [4].

A topic collection regarding the “Clinical Advances in Neuromuscular Diseases: Neurometabolic Disorders” has been edited by Daniela Tavian and myself [5]. We sponsored the Skeletal Muscle Biology in Health and Disease Conference [6] by the University of Florida held in Montegrotto, which was well attended by clinical scientists, including Prof. Gabriele Siciliano (University of Pisa) and Prof. Massimiliano Filosto (University of Brescia that is part of Editorial Board Members). We give special thanks to Prof. Siciliano for organizing the prestigious Pisa Muscle Award recently delivered for scientific careers during a ceremony at Pisa Arsenal Repubblicani, of which I was the recipient. We provided a travel grant for our Editorial Board Member (EBM), Prof. Dr. Ugo Carraro, to promote our journal in the 2023 Padua Days on Muscle and Mobility Medicine [7].

DOAJ indexed *Muscles* in March 2023. 

We held an EBM online meeting in May, and five EBMs joined: Editor in Chief (EiC) Prof. Dr. Corrado Angelini, EBM Prof. Dr. Ugo Carraro, Prof. Dr. Massimiliano Filosto, Prof. Dr. Nathaniel Szewczyk, and Dr. R. Andrew Shanely.

Thanks to all EBMs [8] for their continuous commitment and, in particular, to Dr. Jamie I. Baum (Center for Human Nutrition, USA) who is interested in dietary protein and amino acids for skeletal muscle, Prof. Dr. Marina Bouché (Sapienza University of Rome, Italy), Dr. Gillian Sandra Butler-Browne (Sorbonne Université, France), Prof Guglielmo Duranti (University of Rome, Italy), and all EBMs who contributed to *Muscles* progress in 2023.

In 2023, we had three new EBMs join us: Dr. Kiisa Carla Nishikawa from Northern Arizona University, USA; Prof. Dr. David R. Sinacore from High Point University, USA; and Prof. Dr. Said Hashemolhosseini from Friedrich-Alexander University of Erlangen-Nürnberg, Germany.

## 3. Special Issues

Special Issues hold great significance in representing focus and excellence in journals. They are issues curated around a specific theme or topic, often aiming to shed new light on emerging trends or highlight underrepresented areas within a field. Special Issues offer an opportunity to explore the in-depth new advances of a subject, resulting in a focused compilation of articles that provide valuable insights.

In 2023, there were three Special Issues (SIs) for *Muscles*:

1) Special Issue “Recent Perspectives Regarding Muscle and Exercise Training” (Guest Editor: Dr. Ana Cristina Rodrigues Lacerda) [9].
2) Special Issue “State-of-the-Art Skeletal Muscle Research in the USA” (Guest Editor: Prof. Dr. Nathaniel Szewczyk) [10].
3) Special Issue “Feature Papers in *Muscles*” (Guest Editor: Prof. Dr. Corrado Angelini) [11].

They were instrumental in showcasing unique perspectives, promoting diversity of thought, and advancing specialized areas of research. Special Issues contribute to the overall vibrancy and dynamism of the journal *Muscles*.

We have two new SIs in the works for 2024:

1) Special Issue “Sarcopenia: The Impact on Health and Disease” (Guest Editors: Dr. Nikolaos Papanas and Dr. Nikolaos D. Karakousis) [12].
2) Special Issue “Women’s Special Issue Series: Regulation of Skeletal Muscle Function in Health and Disease” (Guest Editors: Prof. Dr. Louise Deldicque, Dr. Tatiana Kostrominova, and Dr. Jamie I. Baum) [13].

## 4. Challenges and Future Outlook

While the journal *Muscles* has made remarkable strides, certain challenges persist. The articles being submitted and published pose a challenge in terms of maintaining quality control and ensuring rigorous peer-review processes. Additionally, issues related to accessibility and open access continue to grow as the goal of widespread dissemination clashes with the need for long-term sustainability for excellence.

Looking ahead, the future of the journal seems promising. Potential advancements in advanced research in translational myology could aid in improving the process and increasing the efficiency of knowledge dissemination. We opened Twitter to promote all our journal’s news (https://twitter.com/Muscles_MDPI). Additionally, the integration of interactive elements and multimedia content is likely to enhance reader engagement, making the journal more immersive and interactive.

## 5. Conclusions

During 2023, *Muscles* has evolved significantly, moving from traditional publications to an era of numerous reviews and original articles with online accessibility and interactive content. The journal has had a profound impact on the academic and research community, fostering intellectual growth, encouraging scholarly debates, and promoting cross-disciplinary collaboration. Special Issues have played a vital role in highlighting specific themes, advancing specialized research, and providing unique insights.

Despite the challenges, the journal *Muscles* has a promising future, with advancements in technology and evolving reader expectations. The continued growth and success of the journal will undoubtedly shape the landscape of knowledge dissemination for years to come.

## 6. Looking Forward to 2024

The topical section will remain open in 2024, as it enables Guest Editors and Editorial Board Members to invite leading investigators to share their knowledge with our readers. 

Maintaining the high quality of our publications and increasing the visibility of our journal remain essential targets on our road map. It is my pleasure to end this editorial by wishing you all a healthy and prosperous new year. This is also the opportunity for me to warmly thank, our authors, readers, reviewers, scientists of the editorial board, and members of our team in the *Muscles* Editorial Office for their confidence in the journal.
